# A Thermal Tuning Meta-Duplex-Lens (MDL): Design and Characterization

**DOI:** 10.3390/nano10061135

**Published:** 2020-06-08

**Authors:** Ning Xu, Yaoyao Liang, Yuan Hao, Min Mao, Jianping Guo, Hongzhan Liu, Hongyun Meng, Faqiang Wang, Zhongchao Wei

**Affiliations:** 1 Guangdong Provincial Key Laboratory of Nanophotonic Functional Materials and Devices, School of Information and Optoelectronic Science and Engineering, South China Normal University, Guangzhou 510006, China; xuning199405@m.scnu.edu.cn (N.X.); 2018022081@m.scnu.edu.cn (Y.H.); maomin@m.scnu.edu.cn (M.M.); guojp@scnu.edu.cn (J.G.); lhzscnu@163.com (H.L.); hymeng@scnu.edu.cn (H.M.); fqwang@scnu.edu.cn (F.W.); 2 Centre de Nanosciences et de Nanotechnologies, CNRS, Université Paris-Sud—Université Paris-Saclay 10 Boulevard Thomas Gobert, 91120 Palaiseau, France; yaoyao.liang@u-psud.fr

**Keywords:** metalens, duplex, polarization independent, VO_2_ phase change materials

## Abstract

Multifunctional metasurfaces play an important role in the development of integrated optical paths. However, some of the realizations of current multifunctional metasurface devices depend on polarization selectivity, and others change the polarization state of the outgoing light. Here, based on vanadium dioxide (VO_2_) phase change material, a strategy to design a meta-duplex-lens (MDL) is proposed and numerical simulation calculations demonstrate that at low temperature (about 300 K), VO_2_ behaves as a dielectric so that the MDL can act as a transmission lens (transmission efficiency of 87.6%). Conversely, when VO_2_ enters the metallic state (about 355 K), the MDL has the ability to reflect and polymerize electromagnetic waves and works as a reflection lens (reflection efficiency of 85.1%). The dielectric waveguide and gap-surface plasmon (GSP) theories are used in transmission and reflection directions, respectively. In order to satisfy the coverage of the phase gradient in the range of 2π in both cases, we set the antenna as a nanopillar with a high aspect ratio. It is notable that, via symmetrical antennas acting in concert with VO_2_ phase change material, the polarization states of both the incident light and the outgoing light are not changed. This reversible tuning will play a significant role in the fields of imaging, optical storage devices, communication, sensors, etc.

## 1. Introduction

A metasurface [1,2], an artificial composite material with equivalent constitutive parameters synthesized by sub-wavelength structures, exhibits a strong ability to manipulate electromagnetic waves. Compared with traditional optics device, metasurface devices are free of bulky volume and complicated optical path design. The advantages of simple manufacturing, powerful function and easy integration are essential for developing the next generation of integrated optical circuits. There are plenty of theories studying spatial gradient metasurfaces, including but not limited to the theories of dielectric waveguide [3,4], Pancharatnam–Berry phase [5], photonic spin Hall effect (PSHE) [6,7], gap-surface plasmon (GSP) [8,9,10], etc. According to these theories, a number of promising metasurface applications have been proposed, such as metalenses [3,11,12,13], perfect absorbers [14,15], polarization converters [16], and vortex generators [17]. With the deepening of research, single functional metasurfaces have been replaced by versatile and even tunable metasurfaces, which can be realized by utilizing the microelectromechanical system (MEMS) [18], stretchable substrate metasurface [19,20], phase change materials (Ge_2_Se_2_Te_5_ [21,22,23], VO_2_ [24,25,26]), liquid crystal [27] or graphene [28,29,30]. As a thermally sensitive phase change material, VO_2_ is of great function for the reversible phase change in both insulating and metallic states. Exploiting the insulator-metal transition characteristics of VO_2_, lots of novel devices have been proposed, including VO_2_-based absorbers [31,32], tunable-focusing lenses [33], absorber-lenses [34,35], and perfect absorber and polarization converter bifunctional devices [36]. However, part of the multifunctional metasurfaces are implemented by changing the polarization state of the incident light, while the others change the polarization state of outgoing light. The application of multifunctional metasurfaces are restricted by these problems in practical applications.

In this paper, based on VO_2_ phase change material, a general strategy to design a thermal tuning meta-duplex-lens (MDL) in the mid-infrared range (4 µM) is proposed and numerical simulation calculation demonstrated by commercial software FDTD Solutions (Lumerical Inc. in Vancouver, BC, Canada). The designed meta-atoms have the property of realizing transmission and reflection by controlling their temperature. Therefore, in the same structure, transmission or reflection optical focusing can be achieved respectively. At low temperature (about 300 K), VO_2_ enters the insulated state (hereinafter referred to as VO_2_-I). It behaves as a dielectric such that the MDL can deflect and converge electromagnetic waves (transmission efficiency of 87.6%). Conversely, when VO_2_ goes into the metallic state (about 355 K) (hereinafter referred to as VO_2_-M), the MDL could reflect and polymerize electromagnetic waves (reflection efficiency of 85.1%). To achieve this strategy, we use the dielectric waveguide and gap-surface plasmon (GSP) theories in transmission and reflection modes, respectively. In order to satisfy the coverage of the phase gradient in the range of 2π in both cases, we set the antenna as a nanopillar with a high aspect ratio. The innovations of our research are mainly demonstrated in two aspects. One is that, via symmetrical antennas act in concert with VO_2_ phase change material, we do not change the structures of the MDL and the polarization state of the incident light and have no effect on the polarization state of the outgoing light. The other is that, in the same structure, without changing the structural parameters, the active surface temperature control of the metasurface realizes duplex focusing. This device will play an important role in the fields of integrated optical circuit, sensors, optical storage devices, and biological imaging.

## 2. Materials and Methods

**Figure 1:**
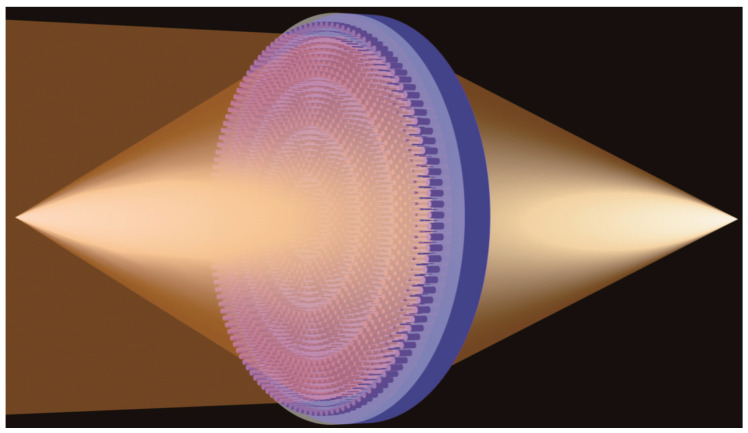
Schematic of duplex focusing of MDL with temperature changes. At room temperature, the metalens achieves transmission focusing (**left**). When the temperature rises to 355 K, VO_2_ becomes the metallic phase and causes the metalens reflection to focus (**right**).

Figure 1 shows the working schematic of a designed device. At room temperature (300 K), the metalens works in transmission mode to focus the incident beam (left). When the temperature rises to 355 K, the metalens works as a reflection metalens (right). It accomplishes the phase modulation in the whole space, which breaks the limitation of the previous metasurfaces that can only operate in half the space. Besides that, it has the property of polarization independence and does not change the polarization state of the outgoing beam.

The essence of the MDL is to design the meta-atoms which could be controlled by temperature to achieve transmission or reflection. These meta-atoms are then combined to cover the phase gradient in whole 2π range. For the purpose, we use the dielectric waveguide and gap-surface plasmon (GSP) theories in transmission and reflection modes, respectively. The application of this design allows our MDL to achieve phase modulation in two directions. In order to satisfy the coverage of the phase gradient in the range of 2π in both cases, we set the antenna as a metal-like nanopillar with high aspect ratio [37]. Since the designed nanopillar antennas are periodically symmetrical, it can achieve polarization-independent focusing in two modes. Therefore, the mode switching is only related to the phase transition state.

### 2.1. Material Model Analysis

VO_2_ has received widespread attention due to its special property of reversible phase change from insulator to metal. The complex permittivity is given by the following well-known dispersion relation [38,39]:

(1) $$\varepsilon \left(\omega \right)=\varepsilon_{\infty }-\frac{\omega_{n}^{2}}{\omega^{2}+i\omega_{c}\omega }+\sum_{i=1}^{n}\frac{s_{i}}{1-\omega^{2}/\omega_{i}^{2}-i\Gamma_{i}\omega /\omega_{i}},$$

**Figure 2:**
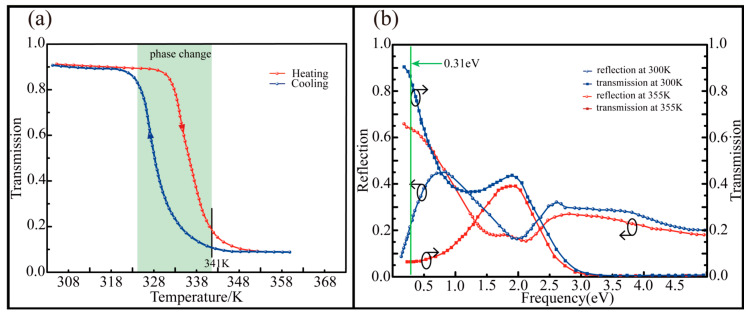
Optical properties of VO_2_ film [39]. (**a**) The transmittance of the VO_2_ film at 4 µM varies with temperature, and the red line and the blue line represent the heating and cooling process, respectively; (**b**) the bi-phase transmittance and reflectance of VO_2_ film in a range of 0.25 eV to 5 eV frequency.

The first term is ultimate high frequency permittivity of VO_2_ material. The second term stands for the contribution of free electron to the dielectric constant. In this term, $\omega_{n}={\left(4\pi n_{c}e^{2}/m^{*}\right)}^{1/2}$ is the carrier density, which is related to the plasma frequency, and

$$\omega_{c}\;=\;e/\mu_{opt}m^{*}$$

is the collision frequency, where $n_{c}$ is the number of conduction electrons and $m^{*}$ is the optical quality of electrons. The summation of Lorentz or classical vibrators is provided by the third term, which considers the non-constant contribution to the remaining dielectric constant within the spectral region of interest. In this term, the lattice vibration intensity, frequency and linewidth are represented by $s_{i}$, $\omega_{i}$, and $\Gamma_{i}$, respectively. The optical properties of VO_2_ film are demonstrated in Figure 2 [39]. Figure 2a depicts the temperature dependence of optical transmission of VO_2_ film at 4 µM. With the increase of temperature, some of the molecules in VO_2_ undergo phase change and diffusion and form a metallic phase structure. The VO_2_ material changes from insulating state to metallic state and the ratio of transmission decreases accordingly. When the critical temperature reaches 341 K, the phase transition is completed. Conversely, the increasing of transmission is caused by the decreasing of temperature, which indicates that the temperature phase transition of VO_2_ is a reversible process. The bi-phase transmission and reflection spectra at 0.25–5 eV (0.248–4.96 µM) are shown in Figure 2b. It is obvious that VO_2_ has a high optical transparency and contrast in the infrared band so that we choose to design and simulate the metasurface at the frequency of 0.31 eV (4 µM).

### 2.2. Dielectric Waveguide Theory

**Figure 3:**
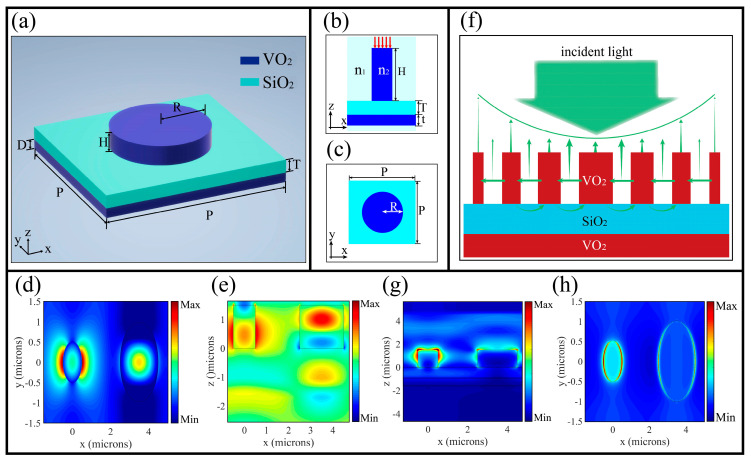
(**a**–**c**) Diagrams of the meta-atom. Along with the longitude, the materials are VO_2_ (*R* = 1.5 µM, *H* = 1.5 µM), SiO_2_ (*T* = 1 µM), VO_2_ (*D* = 0.7 µM) and the lattice constant *p* = 3.5 µM; (**d**,**e**) are the electric near field real $\left(E_{X}\right)$ distribution in the *xy*-plane and *xz*-plane; Left and right pillars have radius of 0.5 µM and 1 µM, respectively; (**f**) The schematic of GSP resonance; (**g**,**h**) The electric near field $real\left|E_{X}\right|$ distribution of VO_2_-M in the *xy*-plane and *xz*-plane. The electric field in the waveguide is coupled with the surface plasma wave to control the phase gradient of the reflected light.

Figure 3a–c illustrate the meta-atoms of the designed metasurface. The unit size of the meta-atom is optimized by parameter sweeps, which is obtained *H* = 1.5 µM, *T* = 1 µM, *D* = 0.7 µM. The lattice constant *p* is chosen to be 3.5 µM to avoid coupling of adjacent waveguides. At room temperature (about 300 K), VO_2_-I is a normal dielectric material with high refractive index (*n*~3) so that it can form an all-dielectric transmission array metasurface. The phase imparted solely by the waveguiding effect is given by [40]:

(2) $$\varphi_{WG}=\beta H=\frac{2\pi }{\lambda_{d}}n_{eff}H,$$

where $n_{eff}$ is the effective index of the fundamental mode ($HE_{11}$) and H (nanocolumns height) is the propagation length. The $n_{eff}$ can be readily computed by a single step-index circular waveguide model. The electric near field $real\left(E_{X}\right)$ distribution in xy-plane and xz-plane are illustrated in Figure 3d,e. Left and right pillars have radius of 0.5 µM and 1 µM, respectively. Most of the electromagnetic wave is trapped in the nano-pillar and unit cell period is optimized to ensure that near-field coupling does not occur in adjacent waveguides.

### 2.3. Gap Surface Plasmon Waveguide Theory

Accompanying with the rise in temperature, VO_2_-M on both sides of the substrate enter the metallic phase to form a metal-insulator-metal (MIM) structure, which composes a metasurface of the reflective array. The schematic diagram is given in Figure 3f. Different from previous metal patches, we apply metal nanocolumns (the height is on the order of microns) to a reflection metasurface for the first time. Due to the fact that high nanocolumns can be equivalent to waveguides, GSP resonance is formed between the two adjacent nanocolumns. Therefore, two adjacent VO_2_ nanopillars and air can be treated as a traditional MIM structure [41]. The surface plasma wave is excited by the evanescent wave at the boundary of the waveguide and the electric field in the waveguide is coupled with the surface plasma wave. The dispersion relation of an MIM obeys the following dispersion equation [42]:

(3) $$\tanh\left(\frac{1}{2}\alpha_{1}d\right)=-\frac{\varepsilon_{1}\alpha_{2}}{\varepsilon_{2}\alpha_{1}},$$

where $\varepsilon_{1}$ and $\varepsilon_{2}$ are the dielectric constants of the insulator and the metal. *d* is the width of the inner insulator. $\alpha_{1}$ and $\alpha_{2}$ are the longitudinal propagation constants in the insulator and metal, respectively. They are related to the effective index of refraction $n_{eff}$ as:

(4) $$\alpha_{i}=\sqrt{n_{eff}^{2}k_{0}^{2}-\varepsilon_{i}k_{0}^{2}},\;\left(i=1,2\right),$$

where $k_{0}=2\pi /\lambda$ is the wave number in a vacuum. Figure 3g,h illustrates the electric field $real\left|E_{X}\right|$ distribution in the *xy*-plane and *xz*-plane. Left and right pillars also have the radius of 0.5 µM and 1 µM, respectively. In this paper, the dielectric layer is equivalent to air. The propagation of light is controlled by changing the spacing between the two nanocolumns, which is equivalent to changing the spacing *d*.

**Figure 4:**
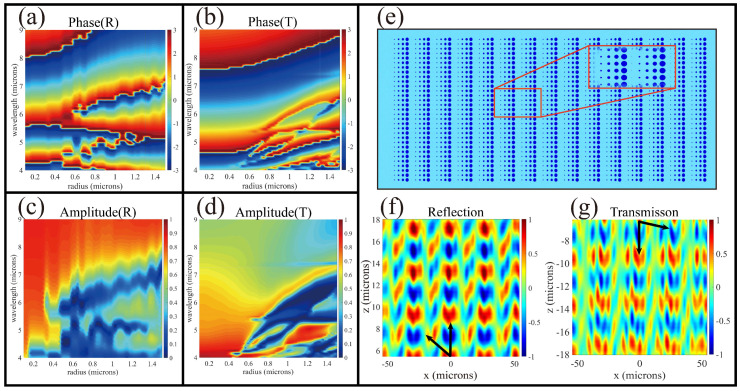
(**a**) Reflection and (**c**) transmission phase response of antennas with varying cylinder radius from 0.1 µM to 1.5 µM for different incidence wavelength from 4 µM to 9 µM. (**b**) Reflection and (**d**) transmission amplitude response of the same antennas. (**e**) The diagram of array structural of beam deflection metasurface; (**f**,**g**) are the electric field (real (Ex)) of reflective and transmissive deflection in the *xz*-plane, respectively.

Figure 4a–d stand for the dependency of utilizing finite difference time domain (FDTD) numerical simulation with phase shift, transmission and reflection on the incidence wavelength. The cylinder cross-section radius are investigated based on commercial software FDTD Solutions (Lumerical Inc., Vancouver, BC, Canada). The radius varies from 0.1 µM to 1.5 µM in steps of 20 nm and the wavelength varies from 4 µM to 9 µM with intervals of 70 nm. Although the phase change state of VO_2_ is switched so that the phase regulated by each antenna is different, the overall trend is roughly consistent with the phase profile of the hyperbolic super lens. Therefore, duplex focusing can be achieved without changing the structure. In order to prove the MDL deflection function to the beam, we first design the metasurface as shown in Figure 4e. According to the generalized Snell’s laws of reflection and refraction [43], the reflection angle and refraction angle can be calculated using:

(5) $$n_{t}sin\theta_{t}-n_{i}sin\theta_{i}=\frac{\lambda_{0}}{2\pi }\frac{d\varphi }{dx},$$

(6) $$sin\theta_{r}-sin\theta_{i}=\frac{\lambda_{0}}{2\pi n_{i}}\frac{d\varphi }{dx},$$

where $\theta_{i}$ is the incidence angle, $\lambda_{0}$ is the working wavelength and $\frac{d\varphi }{dx}$ is the imposed boundary phase discontinuity. Figure 4f,g depicts the electric fields distribution of the transmission and reflection. When the metasurface works in transmission mode (300 K), the device realizes the refraction function. With the temperature increases (355 K), the device enters a reflective state, which accords with the theoretical values quite well. Different from the simulation of one-to-two-period nanopillar arrays that are usually arranged manually with FDTD, this article adopts multiple period antennae and uses generalized Snell’s law to automatically place nanopillars with appropriate radius through scripts. Therefore, the obtained electric field is illustrated in Figure 4f,g and the deflected electric fields of multiple cycles are alternately arranged.

## 3. Concept and Design of Meta-Duplex-Lenses

### 3.1. Design of MDL

**Figure 5:**
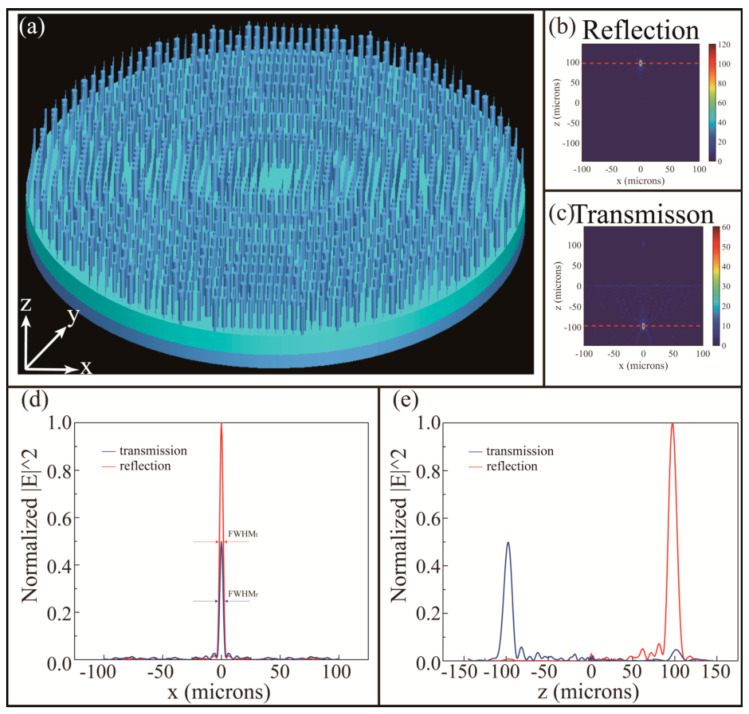
(**a**) The array structural diagram of metalens; (**b**) reflection and (**c**) transmission electric field strength ${\left|E\right|}^{2}$ of far field detection in the *xz*-plane with the working wavelength 4 µM. The position of focus is indicated by red dotted lines. (**d**) Normalized distribution of electric field intensity on the focal plane (*y* = 0) and the arrows indicate the FWHM; (**e**) normalized distribution of the electric field intensity on the propagation plane (*x* = 0).

The components of the metalens are the VO_2_ nanocolumns on the glass substrate (SiO_2_) and the VO_2_ layer below. In order to achieve focus, each nanopillar in position must meet with the requirement of phases below:

(7) $$\varphi \left(x_{i},y_{j}\right)=2\pi n-\frac{2\pi }{\lambda_{0}}\left(\sqrt{x_{i}^{2}+y_{j}^{2}+f^{2}}-f\right),$$

where $x_{i}$ and $y_{j}$ denote the center coordinates of the unit located in the *x*-axis *i*th row and *y*-axis *j*th column, respectively. *f* is the focal length. $\lambda_{0}$ is the operation wavelength of 4 µM and *n* is an arbitrary integer. According to the phase distribution formula given by Equation (7), we design a large-size two-dimensional plane lens. The nanopillar arrangement is displayed in Figure 5a, which has a diameter of 200 µM and an operating wavelength of 4 µM. The parallel light is incident downward along the *z*-axis and the polarization state is linearly polarized light in the x direction. We assume the positive direction is pointing up along the *z*-axis. Both in reflection mode and transmission mode, the designed focal length is 100 µM. With the formula $NA\left(\lambda \right)=D/x\sqrt{f{\left(\lambda \right)}^{2}+D^{2}/4}$, we can calculate the numerical aperture as 0.707.

The far field detection can obtain the electric field strength of the transmission plane (*xz*-plane) as shown in Figure 5b,c so that the MDL is more intuitively displayed. Among them, the red dotted lines is the focus position. Figure 5d,e indicates the normalized electric field intensity ${\left|E\right|}^{2}$ distribution in transmission plane of *x* = 0 and focal plane of *y* = 0, respectively. In both figures, the blue line indicates transmission and the red line indicates reflection. The intensity of the focusing in the transmission mode is attenuated (about 50%) comparing to reflective focusing due to the loss of electromagnetic wave propagation in the three-layer medium. The full width at half maximum (FWHM) in the two working states is calculated from the Figure 5e and indicated by the arrow. The FWHM in transmission mode and reflection mode are 3.286 µM and 3.406 µM, respectively. They are both less than 4 µM in wavelength, which enables the lens to achieve sub-wavelength resolution.

### 3.2. Characterization of Aberration

**Figure 6:**
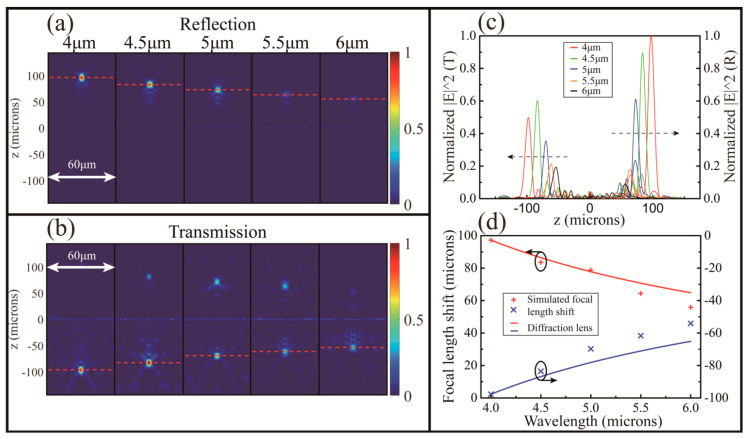
(**a**,**b**) Normalized electric field intensity in the *xz*-plane under different incident wavelengths ranging from 4 µM to 6 µM for MDLs with reflection and transmission, respectively. (**c**) Normalized distribution of electric field intensity of different incident wavelengths on the propagation plane (*x* = 0). (**d**) Simulated positions of focal (symbols) for MDLs with reflection and transmission compared to conventional diffraction lens (lines) at different wavelengths in infrared spectrum.

The metalens above is designed to shape the phase profile for a specific wavelength. In order to characterize the lens performance, it is necessary to study the focuses deviation behaviour of the incident light with a different designed wavelength. We select five wavelengths ranging from 4–6 µM for simulation. These normalized electric field intensities of reflection and transmission in the *xz*-plane are described in Figure 6a,b, respectively.

Here, the focal shift of the diffractive lens is given by:

(8) $$f\left(\lambda \right)=\frac{f\left(\lambda_{0}\right)\lambda_{0}}{\lambda },$$

where λ is the incident wavelength, and $\lambda_{0}$ and $f_{0}$ are the design wavelength and focal length, respectively. Figure 6c is the intensities distribution (in different colors corresponding to their respective wavelengths) of two modes in the *xz*-plane (*y* = 0) at different wavelengths ranging from 4 µM to 6 µM, respectively. All intensities are normalized to the intensities of the design wavelength of reflection mode. Intuitively, the focus intensity tends to decrease as it deviates from the design wavelength. The focal lengths decrease when using a wavelength different to its design value for the MDL (Figure 6d).

We calculate the full width at half maximum in the *y* direction (*x* = 0) on the focal plane in reflection and transmission. The FWHM in transmission mode and reflection mode are 3.999 µM and 3.707 µM, respectively. In order to fully evaluate the performance of the lens, we also analyzed the astigmatism of the lens. The difference in full width at half maximum in the *x* and *y* directions are compared. In reflection, the difference is 0.301 µM, and in transmission, it is 0.713 µM. It can be seen that the imaging quality in reflection mode is better than that in transmission mode, which corresponds to the results obtained in Figure 5 and Figure 6.

In general, transmission efficiency is defined as a ratio of optical power at the focal region on the focal plane to a power of incident light. The power of incident light is equal to the power of light through the aperture of the same size as the lens. Therefore, we can calculate the efficiency in working wavelength are 87.6% (transmission) and 85.1% (reflection), respectively.

## 4. Conclusions

In conclusion, a strategy of designing a thermal tuning meta-duplex-lens (MDL) is presented in this paper. The designed MDL is capable of achieving the duplex focusing of transmission and reflection. The efficiency is 87.6% (transmission) and 85.1% (reflection), respectively. The main difference from previous jobs is that, using VO_2_ phase change material, we do not change the structure and the polarization state of the incident light and have no effect on the polarization state of the outgoing light. The dielectric waveguide and gap-surface plasmon (GSP) theories are used in the forward direction and backward direction, respectively. In order to satisfy the coverage of the phase gradient in the range of 2π in both cases, we set the antenna as a nanopillar with a high aspect ratio, which is the first time to apply metal-like nanopillars in reflection metasurfaces. By adjusting the ambient temperature, the phase change of VO_2_ can be controlled and, consequently, the switching of transmissive and reflective focusing of MDL can be achieved. The phase change of VO_2_ can be induced by a high energy laser, so the MDL that we proposed can be used as a protectable laser lens. When the incident laser energy exceeds the threshold, the transmission light is reflected into the protectable light path so that the device focused by the laser will not be destroyed. Due to the characteristics of duplex transmission and active temperature tuning of MDL, bidirectional optical storage can be realized, increasing the density of optical storage. This reversible tuning device will play a huge role in the field of integrated optical circuit, optical storage devices, sensors and biological imaging.
